# Patient decision aids in breast surgery and breast reconstruction reduce decisional conflict: a systematic review and meta-analysis

**DOI:** 10.1007/s10549-025-07752-0

**Published:** 2025-06-30

**Authors:** Tokoya Williams, Keenan Fine, Emily Duckworth, Tarifa Adam, Caden Bozigar, Annie McFarland, Antoinette Nguyen, Brigid M. Coles, Robert D. Galiano

**Affiliations:** 1 Loyola University Chicago College of Arts and Sciences, Chicago, IL USA; 2 Northwestern University Feinberg School of Medicine, 259 East Erie Street, Suite 2060, Chicago, IL 60611 USA; 3 University of South Carolina School of Medicine Greenville, Greenville, SC USA; 4 University of Illinois College of Medicine, Chicago, IL USA; 5 University of Rochester School of Medicine and Dentistry, Rochester, NY USA

**Keywords:** Breast cancer, Breast surgery, Breast reconstruction, Patient decision aids

## Abstract

**Purpose:**

Around 310,000 new cases of breast cancer (BC) are diagnosed each year. Complex treatment options often overwhelm patients. Patient decision aids (PDAs) assist in surgical decision-making, but reviews of their quality and efficacy are limited. This study systematically reviews breast surgery (BS) and breast reconstruction (BR) PDAs using the International Patient Decision Aid Standards and Cochrane tools to identify gaps and provide evidence-based recommendations.

**Methods:**

A systematic review following PRISMA guidelines examined the impact of PDAs on decision-making for BC patients considering BS and BR. From 1198 articles, 35 met the inclusion criteria. Data on PDA components, study design, and results were extracted, focusing on decisional conflict and anxiety, measured by the Decisional Conflict Scale (DCS) and the State-Trait Anxiety Inventory (STAI). PDA quality and study design were assessed using Cochrane, IPDASi, and ROBINS-I tools.

**Results:**

Eight studies evaluated the effect of PDAs on decisional conflict. The pooled mean difference of 3.08 points (95% CI: − 0.62 to 6.79, *p* = 0.10) favored the PDA group but was not statistically significant. Two studies, however, reported notable reductions in decisional conflict with effect sizes of 13.50 and 12.80 points, respectively. The pooled effect size of PDA exposure on patient anxiety was 1.93 (95% CI: − 0.46 to 4.31) in favor of PDAs, but was not statistically significant (*p* = 0.11). The evaluation of PDA content quality revealed variable results.

**Conclusion:**

BS and BR PDAs were not found to significantly reduce decisional conflict and anxiety in breast cancer patients. Standardized, evidence-based tools are needed.

## Introduction

Approximately 310,000 new cases of invasive breast cancer are diagnosed every year in the United States and account for nearly 30% of all new female cancers each year [1]. Breast surgery (BS) and breast reconstruction (BR) are vital components of the recovery and treatment process for individuals affected by breast cancer. Vast advancements in treatment and surgical options have paved the way for higher survival rates and an overall better quality of life. As the quality of treatment improves, the complexity of available treatment options also escalates. Navigating such a multitude of options proves to be extremely daunting and complex for many individuals. Along with this complexity, patients often feel anxious and overwhelmed with the amount of new information they are presented with, which may negatively impact postoperative satisfaction and overall quality of life [2]. Additionally, it is common for many individuals to experience conflict with their decisions, especially if they have trouble pinpointing their values and goals with their care provider [3]

Patient decision aids (PDAs) have been constructed to assist patients in their decision-making process by providing valuable information, support, and direction in their breast cancer surgery journey. PDAs are provided in many forms for both individual and consultative purposes, including pamphlets, apps, interactive websites, and even personalized counseling sessions [4]. These aids aim to provide patients with a necessary understanding of surgical options, recovery expectations, and long-term outcome predictions. PDAs serve as a middle ground to facilitate helpful learning for both the patient and the healthcare team assisting the patient along their journey to ensure that patients feel confident and well-informed in their choices.

Previous BR PDAs have shed light on the tools necessary for understanding the reconstructive process and possibilities [5]. However, there is a paucity of complete and comprehensive reviews of both BS and BR PDAs. This absence comes from certain factors such as the lack of standardization from the many forms of PDAs, patient-specific factors, and subjectivity within assessments. A full review with both the Cochrane assessment tool and the IPDASi provides an efficient and standardized approach to assess various studies and critically analyze them to identify bias to provide a more thorough evaluation of the current BS and BR PDAs. Addressing these gaps in BR and BS literature is vital and cannot be overstated. Through an extensive examination of current literature on BS and BR PDAs, we aim to summarize and offer a comprehensive understanding of their content quality, the rigor of studies evaluating their efficacy, and their overall impact. Our primary focus is on whether these PDAs effectively reduce patient anxiety and decisional conflict. We hope to identify potential opportunities for improving the overall quality of BS and BR patient decision aids.

## Methods

**Fig. 1:**
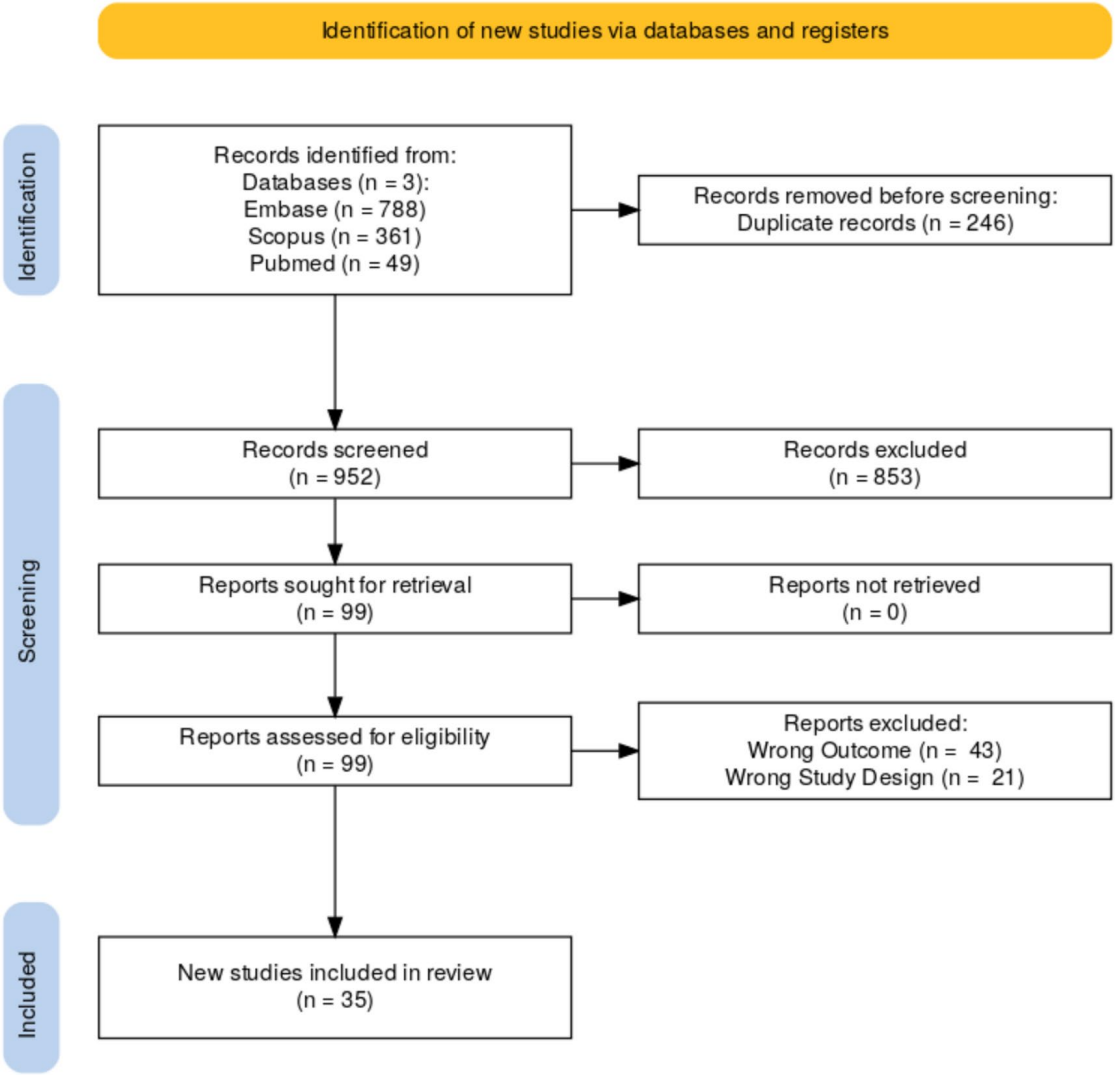
PRISMA

This systematic review was conducted following the Preferred Reporting Items for Systematic Reviews and Meta-Analyses (PRISMA) (Fig. 1) guidelines. The eligibility criteria included studies published in peer-reviewed journals focusing on patients diagnosed with breast cancer who were given a patient decision aid to guide and assist their decision regarding breast cancer surgery and breast reconstruction. Included studies also had to provide quantitative data on the impacts of PDA usage on patient decision-making. All forms of decision aids were considered, including paper, digital, pre- or post-consultation administration, etc. Non-peer-reviewed articles, such as case reports, reviews, editorials, and commentaries, were excluded.

We conducted a comprehensive search of Cochrane, Embase, and PubMed databases for articles published up to June 2024. The following search terms were used: “breast cancer”, “breast reconstruction surgery”, “decision aid”, “decision making”, and “decision support,” Our PubMed search yielded 361 results with the query, (“breast cancer” OR “breast neoplasm”) AND (“breast reconstruction”) AND (“decision aid” OR “decision making” OR “decision support”). Embase yielded 788 results with (‘breast cancer’/exp OR ‘breast neoplasm’/exp) AND ‘breast reconstruction’ AND (‘decision aid’/exp OR ‘decision making’/exp OR ‘decision support’/exp). Cochrane yielded 46 trials & 3 reviews using “breast cancer” AND “breast reconstruction” AND (“decision aid” OR “decision making” OR “decision support”). If an article described the use of an already existing PDAs described elsewhere, we found the original paper explaining the PDAs to gather data for extraction.

A total of 1198 articles were imported into Rayyan (Qatar Computing Research, Doha, Qatar) for screening, and 227 duplicates were removed. A total of seventy-one articles were screened by two blinded reviewers for title and abstract. A third independent reviewer resolved conflicts. Based on the title and abstract screening, 83 articles were included. After full-text screening, a total of 35 papers were selected for inclusion. Ten articles reported on the use of a breast surgery decision aid, including the option of mastectomy and no reconstruction or breast-conserving surgery. Nineteen papers presented findings based on PDAs specifically designed to help patients choose a specific type of reconstruction. Three articles, Varelas 2020, Fang 2021, and Lin 2021, did not explain the surgical options discussed in the PDAs. The 19 articles that specified reconstructive surgical options presented an array of procedures, including implant-based, autologous, delayed, and immediate, as well as specific flap types like latissimus or DIEP.

Data extraction was performed by six reviewers who collected information on study design, PDAs components and outcomes, and PDAs quality assessment. Studies were reviewed in Rayyan, and relevant data were transcribed by reviewers into a series of Excel sheets. Each study’s data were extracted by a single reviewer. Study design data included the following: study type, participant characteristics, source of sample, specific PDAs, number of control and PDAs-exposed participants, mean age of participants, and mean follow-up.

The ROBINS-E tool was used to determine the risk of bias (ROB) for all included texts to determine the strength and each study’s findings (Table 3) [6]. Domains one through seven assessed the presence of confounding variables, exposure measurement, participant selection, post-exposure interventions, missing data, outcome measurement, and results reporting, respectively. Outcomes of interest for the meta-analysis included the decisional conflict scale (DCS) and the State-Trait Anxiety Inventory (STAI). Decisional conflict is defined as uncertainty about a course of action. The scale is based on five sub-scales that measure an individual’s uncertainty in making health-related decisions, reflecting factors like perceived risks, benefits, and support. A higher score on the DCS is indicative of higher decisional conflict [7]. The STAI scale contains 20 items for assessing trait anxiety and 20 for state anxiety. State anxiety items include: “I am tense; I am worried” and “I feel calm; I feel secure.” Trait anxiety items include: “I worry too much over something that really doesn’t matter” and “I am content; I am a steady person.” All items are rated on a 4-point scale. Higher scores indicate greater anxiety [8].

The Cochrane PDA assessment evaluates PDA quality based on whether the tool explicitly states the decision that needs to be made and helps the user to identify their values and preferences [9]. The Cochrane assessment also evaluates whether the decision aid clearly states the risks and benefits of each surgical option, including the specific probability of encountering these risks. The International Patient Decision Aid Standards Instrument (IPDASi) was utilized to assess the quality of our included PDAs based on four domains related to information about surgical options, outcome probabilities, values clarification, and decision guidance [10, 11].

The ROBINS-E, the Cochrane, and the IPDASi were each assessed by two independent evaluators. A narrative synthesis was performed to summarize the findings of each paper, supplemented by tables and figures to provide a clear and concise overview of the current literature.

## Results

### Decisional conflict scale

**Fig. 2:**
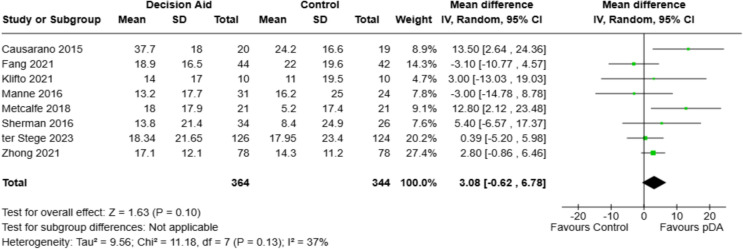
Decisional Conflict Scale Forest Plot vs. SOC

Eight studies were included in the meta-analysis evaluating the decisional conflict scale outcome, all providing BS or BR pre-PDA and post-PDA use means and standard deviations necessary for estimating effect sizes (Fig. 2). Six of the studies reported a follow-up time for the post-PDA assessment of DCS, ranging from one week to 12 months. A total of 364 patients were in the PDA groups, while 344 were in the standard of care (SOC) control groups. The analysis yielded an *I*^*2*^ = 37%, permitting a pooled analysis using a random-effects model. The pooled mean difference was 3.08 points (95% CI: − 0.62 to 6.79, *p* = 0.10), favoring the PDAs group, though not statistically significant. Notably, two studies, Causarano (2013) and Metcalfe (2018), showed significant improvements in the PDA groups, with effect sizes of 13.50 (95% CI: 2.64 to 24.36) and 12.80 (95% CI: 2.12 to 23.48), respectively. The remaining six studies reported no significant differences between the PDAs and SOC groups.

### State-trait anxiety inventory (STAI)

**Fig. 3:**
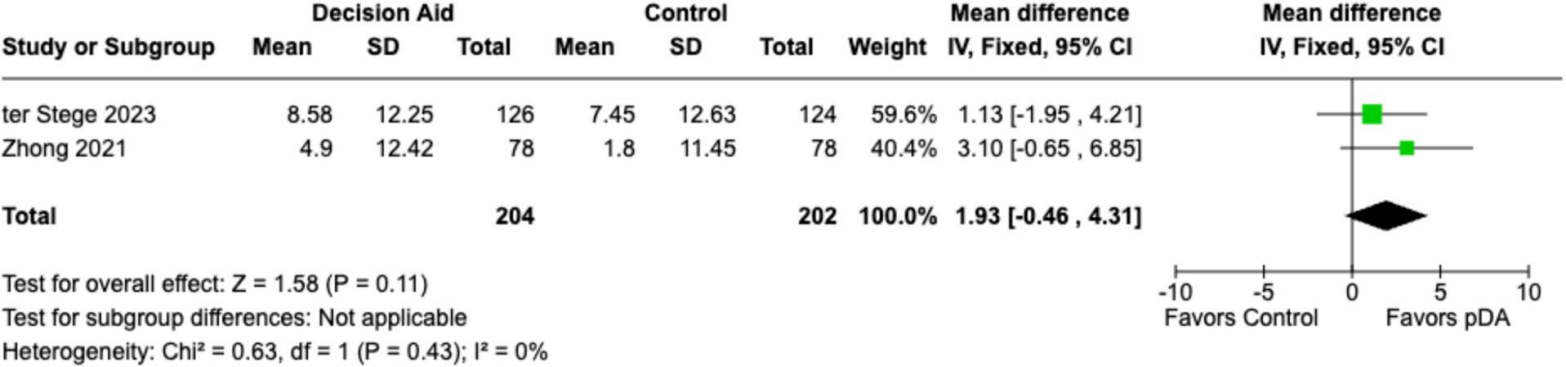
State-Trait Anxiety Inventory Plot vs. SOC

Two studies, Ter Stege et al. (2023) and Zhong et al. (2021), were included in the meta-analysis using STAI as an outcome measure (Fig. 3). A total of 204 PDA patients and 202 SOC patients were included. The overall effect size was 1.93 (95% CI: − 0.46—4.31) in favor of the PDAs, although it was insignificant (*p* = 0.11).

### Risk of bias

All studies were assessed as having a low risk of bias (Table 1). The papers included in this review had the greatest likelihood of exhibiting bias in domains three and five due to potential disparities in participant demographics in control and experimental groups, as well as a lack of complete participation at all time points in the study. However, Domains 1–4 and 6–7 had no risk of bias, while domain 5 had a small risk of bias.

**Table 1.**
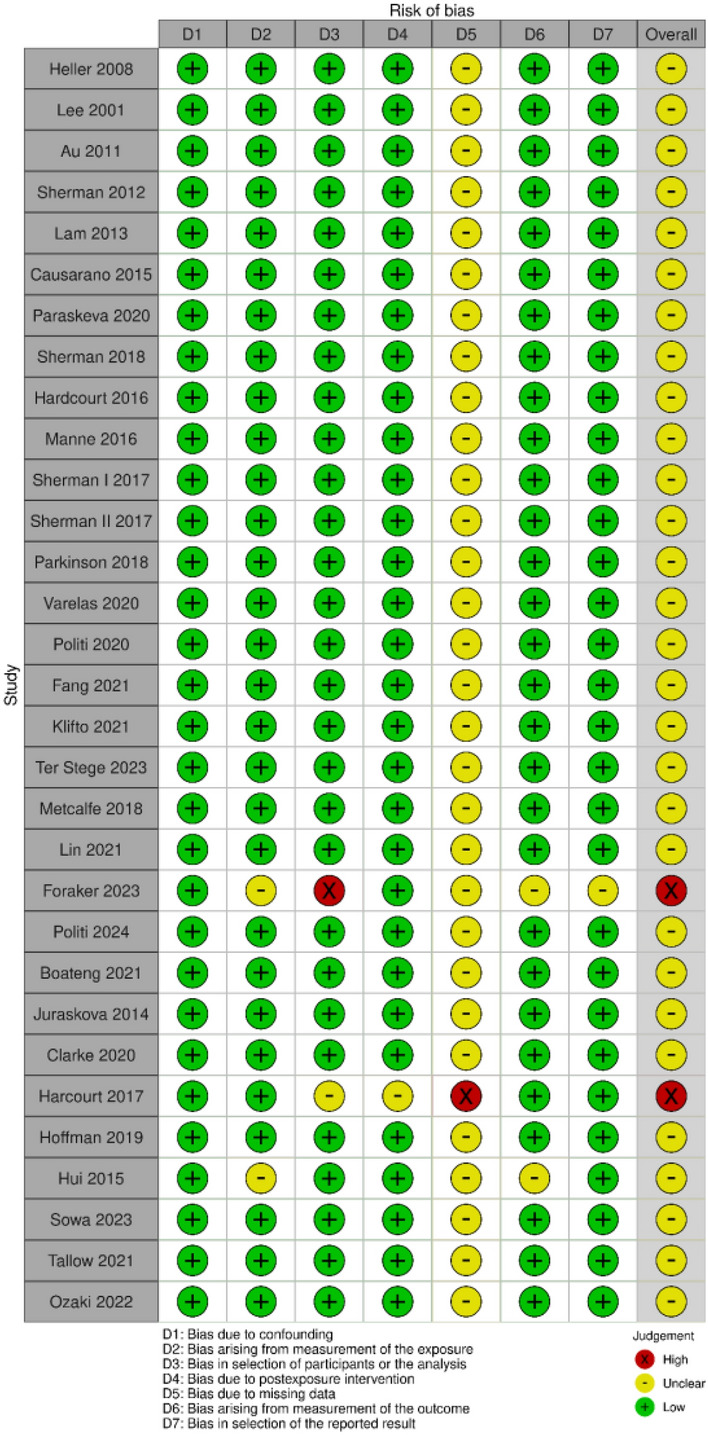
Risk of Bias (RoB)

### PDA content quality

Eight studies did not meet at least one of the three domains of the Cochrane PDA content quality assessment; all eight specifically failed to provide evidence-based information on key elements such as the condition, benefits, harms, probabilities, and scientific uncertainties of BS/BR (Tables 2 and 3). Most studies demonstrated strong performance across the four key domains of the IPDASi content quality assessment (Table 4). However, a few studies showed opportunities for improvement in the ‘Probabilities’ domain (Heller et al., Foraker et al., Harcourt et al., Clarke et al.).ss

**Table 2:** Cochrane Assessment of Breast Cancer Surgery pDAs

Author/yr	Article title	Decision aid type: breast cancer surgery (BC) or breast reconstruction surgery (BR)	Does the decision aid explicitly state the decision that needs to be made?	Does the decision aid provide evidence-based information on condition, benefits, harm, probabilities, and scientific uncertainties of BS/BR?	Does the decision aid help the patient clarify their values based on the potential benefits and harms of BS/BR?
Au 2011	Development and pilot-testing of a Decision Aid for use among Chinese women facing breast cancer surgery	BC	Yes	Yes	Yes
Hawley 2016	Evaluating a Decision Aid for Improving Decision Making in Patients with Early Stage Breast Cancer	Both	Yes	Yes	Yes
Lam 2013	Reducing treatment decision conflict difficulties in breast cancer surgery: a randomized controlled trial	BC	Yes	Yes	Yes
Manne 2016*	Understanding decision-making for and against oncoplastic breast-conserving surgery as an alternative to a mastectomy in early breast cancer: UK ANTHEM qualitative study	BC	No	No	No
Ozaki 2022	Satisfaction survey on a preoperative explanation method using three-dimensional breast imaging for breast cancer patients considering breast-conserving surgery	BC	No	No	No
Parkinson 2018	Cost-effectiveness of the BRECONDA decision aid for women with breast cancer: results from a randomized controlled trial	BC	Yes	Yes	Yes
Sherman 2017	Facilitating decision-making in women undergoing genetic testing for hereditary breast cancer: BRECONDA randomized controlled trial results	BC	Yes	Yes	Yes

**Table 3:** Cochrane Assessment of Breast Reconstruction pDAs

Author/yr	Article title	Decision aid type: breast cancer surgery (BC) or breast reconstruction surgery (BR)	Does the decision aid explicitly state the decision that needs to be made?	Does the decision aid provide evidence-based information on condition, benefits, harm, probabilities, and scientific uncertainties of BS/BR?	Does the decision aid help the patient clarify their values based on the potential benefits and harms of BS/BR?
Boateng 2021	Implementing an Electronic Clinical Decision Support Tool Into Routine Care: A Qualitative Study of Stakeholders’ Perceptions of a Post-Mastectomy Breast Reconstruction Tool	BR	Yes	Yes	Yes
Causarano 2015	Preconsultation educational group intervention to improve shared decision-making for postmastectomy breast reconstruction: a pilot randomized control trial	BR	Yes	Yes	Yes
Clarke 2020	PEGASUS: the Design of an Intervention to Facilitate Shared Decision-making in Breast Reconstruction	BR	Yes	No	Yes
Fang 2021	Long-Term Effectiveness of a Decision Support App (Pink Journey) for Women Considering Breast Reconstruction Surgery: pilot Randomized Controlled Trial	BR	No	No	No
Foraker 2023	Using the sociotechnical model to conduct a focused usability assessment of a breast reconstruction decision tool	BR	Yes	Yes	Yes
Harcourt 2017	A study protocol of the effectiveness of PEGASUS: a multi-centred study comparing an intervention to promote shared decision making about breast reconstruction with treatment as usual	BR	Yes	No	Yes
Hardcourt 2016	The acceptability of PEGASUS: an intervention to facilitate shared decision-making with women contemplating breast reconstruction	BR	Yes	No	Yes
Hawley 2016	Evaluating a Decision Aid for Improving Decision Making in Patients with Early Stage Breast Cancer	Both	Yes	Yes	Yes
Heller 2008	Interactive digital education aid in breast reconstruction	BR	No	Yes	No
Hoffman 2019	Considering Breast Reconstruction after Mastectomy: A Patient Decision Aid Video and Workbook	BR	Yes	No	Yes
Hui 2015	Design and Focus Test of a Preconsultation Decision Aid for Breast Cancer Reconstruction Patients: A Quality Improvement Initiative	BR	Yes	Yes	Yes
Juraskova 2014	Improving preparedness prior to reconstructive breast surgery via inclusion of 3D images during pre-operative counselling: a qualitative analysis	BR	No	No	No
Klifto 2021	Decision aid for women with newly diagnosed breast cancer seeking breast reconstruction surgery: a prospective, randomized, controlled, single-blinded, pilot study	BR	Yes	Yes	Yes
Laun 2016	Effects of a novel decision aid for breast reconstruction: a randomized prospective trial	BR	Yes	Yes	Yes
Lee 2010	Computer-based learning module increases shared decision making in breast reconstruction	BR	Yes	Yes	Yes
Lin 2021	Development and Usability Testing of a Decision Support App for Women Considering Breast Reconstruction Surgery	BR	Yes	Yes	Yes
Manne 2016	Acceptability and pilot efficacy trial of a web-based breast reconstruction decision support aid for women considering mastectomy	BR	Yes	Yes	Yes
Metcalfe 2018	Development and testing of a decision aid for women considering delayed breast reconstruction	BR	Yes	Yes	Yes
Paraskeva 2020	PEGASUS: the effectiveness of an intervention designed to promote shared decision making about breast reconstruction	BR	Yes	No	Yes
Politi 2020	A Randomized Controlled Trial Evaluating the BREASTChoice Tool for Personalized Decision Support About Breast Reconstruction After Mastectomy	BR	Yes	Yes	Yes
Politi 2024	A Randomized Controlled Trial of the Implementation of BREASTChoice, a Multilevel Breast Reconstruction Decision Support Tool With Personalized Risk Predictiona	BR	Yes	Yes	Yes
Sherman 2012	Breconda: development and acceptability of an interactive decisional support tool for women considering breast reconstruction	BR	Yes	Yes	Yes
Sherman 2016	Reducing Decisional Conflict and Enhancing Satisfaction with Information among Women Considering Breast Reconstruction following Mastectomy: results from the BRECONDA Randomized Controlled Trial	BR	Yes	Yes	Yes
Sherman 2017	Qualitatively understanding patients’ and health professionals’ experiences of the BRECONDA breast reconstruction decision aid	BR	Yes	Yes	Yes
Ter Stege 2023*	Efficacy of a decision aid in breast cancer patients considering immediate reconstruction: results of a randomized controlled trial	BR	Yes	Yes	Yes

**Table 4:**
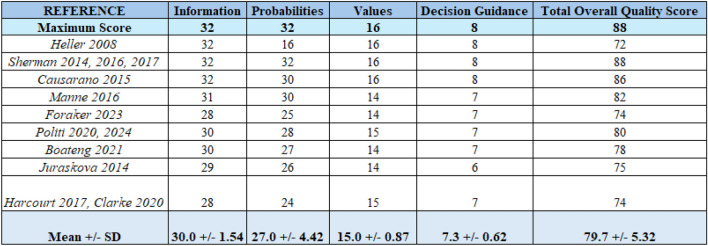
Assessment of Patient Decision Aid Quality Using the International Patient Decision Aid Standards Instrument (IPDASi). This table presents the quality assessment of fully accessible patient decision aids using the IPDASi criteria. Each decision aid was evaluated based on key domains of the IPDASi framework: Information, Probabilities, Values, and Decision Guidance

*Information*: Provides information about options in sufficient detail for decision making

*Probabilities*: Presents probabilities of outcomes in an unbiased and understandable way

*Values*: Includes methods for clarifying and expressing patients’ values

*Decision* *Guidance*: Includes structured guidance in deliberation and communication

## Discussion

Our review of breast reconstruction PDAs builds upon prior research by addressing critical gaps in the existing literature. Unlike a previously published study, which assessed decisional conflict and anxiety scores at a single time point post-exposure to a breast reconstruction decision aid [7], our investigation evaluates these outcomes both before and after exposure. This approach allowed us to assess the degree of change attributable to the intervention. The result is a more robust analysis by incorporating effect size, offering a clearer understanding of the impact of decision aids on patient experience. Additionally, while the prior study focused solely on decision aids for breast reconstruction [7], our research expands the scope to include decision-making for both breast cancer surgery and breast reconstruction surgery. This broader perspective provides nuanced insights into the decision-making process, making our findings uniquely valuable to improving patient-centered care and optimizing surgical outcomes.

While the results of this study suggest a trend towards reduced decisional conflict with the use of both BS and BR PDAs compared to without, contrary to the Su et al. findings, we did not find the change in decisional conflict scores before and after PDAs exposure to be statistically significant. The former led us to dissect the potential reasons for the varied performance levels of the respective PDAs, with a focus on the study characteristics consistent with the PDAs that had the largest positive effect on decisional conflict and patient anxiety.

Both Causarano et al. and Metcalfe et al. demonstrated the largest effect size on decisional conflict after PDA exposure [12, 13]. The strengths of the Causarano et al. PDAs are that the intervention was a 2 hour, in-person workshop hosted by medical staff, which allowed participants to ask clarifying questions in real-time. Though the Metcalfe et al. PDA is web-based, similar to Causarano et al., the PDA’s design is interactive, thereby allowing the tool to adapt to individual patient profiles, preferences, and health conditions to provide personalized recommendations [13]. Notably, both decision aid tools were constructed based on the *Ottawa Decision Support Framework* [14], which incorporates psychological and social aspects of patient decision-making to guide practitioners in the design of interventions that adequately equip patients with the information they need to make high-quality decisions. Additionally, both tools demonstrated top performance in all three domains of the Cochrane review. In line with the former, the studies with the lowest impact on decisional conflict (Fang et al. and Manne et al.) failed all three components of the Cochrane assessment [15, 16]. The former suggests that the use of evidence-based information, explicitly stating the decision that the patient needs to make, and incorporation of interactive components of the PDAs that assist the patient with clarifying her values are of paramount importance in the design of an effective breast reconstruction surgery decision tool. Both tools accomplished the former by breaking the decision into four key domains: the desired natural appearance of their breasts, the preferred length of hospital stay, whether they wish to avoid implanting foreign materials, and the importance of minimizing the number of surgeries. Patients were guided to rank these priorities, allowing them to focus on what mattered most to them. This structured approach highlights why both tools were so effective. Overall, the superior effect size of both of the aforementioned studies is consistent with previously published studies, which have shown that when surgical patients are provided with the information necessary to participate in shared decision-making, they are more satisfied with their outcomes and experience less regret regarding their choices [17–19].

Anxiety assessment was largely limited by a low number of studies (ter Stege et al. and Zhong et al.) [20, 21]. Though not statistically significant, overall, the effect size on anxiety favored the PDAs over the standard of care. A breast cancer diagnosis carries a significant risk of psychological distress for patients. Nearly 50% of women diagnosed with breast cancer experience depression, anxiety, or both [22–25]. In part, this lower quality of life results from the effects of medical treatment. Roughly 40% of those affected by breast cancer undergo mastectomy [26]. The effects of breast removal often cause negative feelings regarding body image, particularly for young women [27, 28]. Given the former, the results of this study highlight the need for more large-scale, randomized control trials that focus on the psychological impact of breast reconstruction decision aid tools.

The IPDASi framework, which evaluates the quality of patient decision aids (PDAs), emphasizes the importance of presenting probabilities of outcomes in a clear, unbiased, and comprehensible way [10]. Interestingly, when comparing this with the Cochrane assessment, which also includes multiple domains of evaluation, a consistent issue emerges: while most studies demonstrated proficiency across the content quality domains, the results of this study highlight that many BS and BR PDAs tend to fall short in offering patients precise, personalized information about their risks, benefits, and potential outcomes. This gap is particularly evident in how these aids handle the presentation of outcome probabilities. While patients are expected to make informed decisions, the quality of content often does not provide them with the tailored information they need to understand the likelihood of different outcomes. This lack of clarity and precision undermines the core purpose of decision aids: empowering patients with the knowledge they need to make choices that are best suited to their circumstances. Both the Cochrane and IPDAS assessments reveal a consistent shortcoming in PDAs—the failure to adequately communicate tailored probabilities and outcomes, which is crucial for effective patient decision-making. A key feature of the higher-performing studies in this category is the interactive nature of the decision aid, which incorporated the individual patient’s medical history into the predicted risk of post-surgical complications (Causarano et al., Manne et al., Sherman et al. 2014 and 2016, Politi et al. 2020 and 2024) [12, 16, 29–32]. This tailored approach stands in contrast to decision aids based on group information sessions about breast surgery and breast reconstruction, highlighting a potential advantage of computer-based tools. The individualized, private nature of the computer-based approach may create a more conducive environment for patients to share sensitive medical history, ultimately enhancing the accuracy and relevance of the risk predictions.

Notably, the study with the highest effect size on decisional conflict was designed as a randomized control trial, with 95% retention for each outcome time point [12]. This underscores the critical importance of conducting Level I randomized controlled trials and designing studies that prioritize patient retention and minimize loss to follow-up, ensuring the reliability and impact of the findings. The studies that demonstrated a high risk of bias [33, 34] were excluded from the data synthesis as they did not report the baseline (pre-intervention) decisional conflict and anxiety scores necessary for comparison with post-intervention outcomes following the decision aid implementation. Both studies demonstrated bias in the areas of participant selection and missing data. This highlights the importance of implementing mechanisms to enhance study participant retention and ensure a diverse study population, which are critical for optimizing study design to effectively evaluate the efficacy of a patient decision aid. Breast reconstruction surgery improves the overall quality of life in breast cancer survivors [35, 36]. Despite this benefit, white women who undergo breast reconstruction surgery consistently report higher satisfaction, quality of decision, surgical access, and informational access compared to their Non-White peers [35–37]. Further, Black and Hispanic women are particularly vulnerable to postoperative complications, which contributes to poorer patient-reported satisfaction with their aesthetic results compared to white women [38–42]. Taken together, the need for more racially inclusive, large-scale, randomized control trials that evaluate breast reconstruction decision aids is clear.

### Limitations

While our study improves and expands upon previous work, there are limitations to the present investigation. Inter-rater reliability was not assessed during the review process, potentially impacting the validity and reliability of our findings. Furthermore, scoring with the IPDASi may involve some subjectivity due to the broad scope of the items. To enhance the reliability of our quality evaluation, we implemented two rounds of scoring for each assessment tool. Importantly, the studies included in the review include a wide range of patient populations, types of surgeries, interventions, and outcome measures. This variability can make it difficult to synthesize results accurately and meaningfully. Further, PDAs in BS and BR may not always be rigorously tested in randomized controlled trials (RCTs), which could limit the quality of evidence available for meta-analysis. Many studies may rely on observational data, which are more prone to biases. Finally, cultural, social, and healthcare system differences between study populations can influence how decision aids are implemented and received by patients. These factors may limit the generalizability of findings to broader populations. Despite these limitations, we believe the findings of this study provide valuable insights for improving decision aids and potentially enhancing their effectiveness.

## Conclusions

**Fig. 4:**
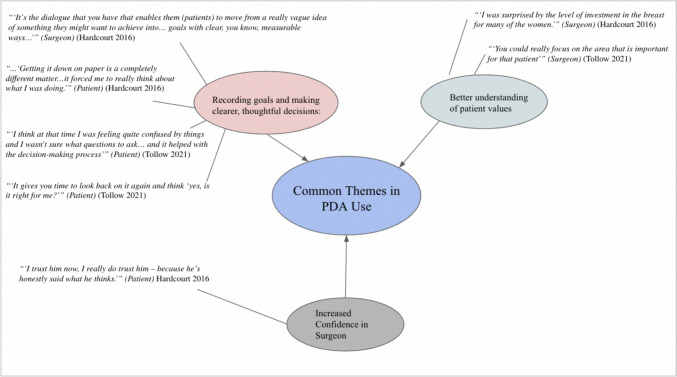
Patient and Surgeon-reported experiences regarding pDA use. Articles pertaining to pDA use and Breast Cancer Surgery were evaluated based on the Cochrane Recommendations for Patient Decision aid Assessment (citation):* Does the decision aid explicitly state the decision that needs to be made?; Does the decision aid provide evidence-based information on the condition, benefits, harm, probabilities, and scientific uncertainties of BS/BR?; Does the decision aid help the patient clarify their values based on the potential benefits and harms of BS/BR?*

Although the results were not statistically significant, the overall trend toward a positive effect of PDAs on decisional conflict and patient anxiety suggests that these tools hold great promise. However, there is room for improvement in the design of PDAs, particularly in ensuring the consistent incorporation of evidence-based information and including interactive components that help patients clarify their preferences. Additionally, the study design needs to improve by including both baseline (pre-intervention) and post-intervention scores to evaluate decisional conflict and patient anxiety, as well as the impact of decision aids on these outcomes. Enhancing diversity—especially racial diversity—and improving patient retention are also critical to maximizing the power of the sample size. Finally, there is a pressing need to focus on the psychological impact of PDAs, given the high rates of depression and anxiety associated with mastectomy, as well as the significant positive psychological effects of breast reconstruction after mastectomy (Fig. 4). Several studies have called for BS and BR educational outreach to underserved communities, and the implementation of tailored patient-level interventions that promote equity in surgical care experiences [49,50]. Other studies have called for increased availability of language and culturally concordant educational materials that ultimately work toward eliminating these disparities.

## Data Availability

The datasets analyzed during this study are available publicly on PubMed, Embase, and Cochrane.
